# Ischemic Preconditioning Protects against Spinal Cord Ischemia-Reperfusion Injury in Rabbits by Attenuating Blood Spinal Cord Barrier Disruption

**DOI:** 10.3390/ijms140510343

**Published:** 2013-05-17

**Authors:** Bo Fang, Xiao-Man Li, Xi-Jia Sun, Na-Ren Bao, Xiao-Yan Ren, Huang-Wei Lv, Hong Ma

**Affiliations:** 1 Department of Anesthesiology, First Affiliated Hospital, China Medical University, Shenyang 110001, Liaoning, China; E-Mails: drunk0630@yahoo.com.cn (B.F.); xijia2000@126.com (X.-J.S.); naren1980@126.com (N.-R.B.); xiaoyanren1983@126.com (X.-Y.R.); lvhuangwei@126.com (H.-W.L.); 2 Key Laboratory of Medical Cell Biology, Ministry of Education, China Medical University, Shenyang 110001, Liaoning, China; E-Mail: disneyer@hotmail.com

**Keywords:** ischemic preconditioning, ischemia-reperfusion injury, spinal cord, blood spinal cord barrier

## Abstract

Ischemic preconditioning has been reported to protect against spinal cord ischemia-reperfusion (I-R) injury, but the underlying mechanisms are not fully understood. To investigate this, Japanese white rabbits underwent I-R (30 min aortic occlusion followed by reperfusion), ischemic preconditioning (three cycles of 5 min aortic occlusion plus 5 min reperfusion) followed by I-R, or sham surgery. At 4 and 24 h following reperfusion, neurological function was assessed using Tarlov scores, blood spinal cord barrier permeability was measured by Evan’s Blue extravasation, spinal cord edema was evaluated using the wet-dry method, and spinal cord expression of zonula occluden-1 (ZO-1), matrix metalloproteinase-9 (MMP-9), and tumor necrosis factor-α (TNF-α) were measured by Western blot and a real-time polymerase chain reaction. ZO-1 was also assessed using immunofluorescence. Spinal cord I-R injury reduced neurologic scores, and ischemic preconditioning treatment ameliorated this effect. Ischemic preconditioning inhibited I-R-induced increases in blood spinal cord barrier permeability and water content, increased ZO-1 mRNA and protein expression, and reduced MMP-9 and TNF-α mRNA and protein expression. These findings suggest that ischemic preconditioning attenuates the increase in blood spinal cord barrier permeability due to spinal cord I-R injury by preservation of tight junction protein ZO-1 and reducing MMP-9 and TNF-α expression.

## 1. Introduction

Spinal cord ischemia-reperfusion (I-R) can result in debilitating injuries to the central nervous system, including immediate or delayed paraplegia [1]. It is a major complication of surgery for thoracic and thoraco-abdominal aneurysms with a reported incidence of 3%–18% [2]. Many strategies have been developed to increase ischemic tolerance in the spinal cord, including partial bypass, drainage of the cerebrospinal fluid, pharmacological interventions, and hypothermia. Unfortunately, despite these advances, the incidence of paraplegia remains high and poses a persistent and devastating threat to patients [3].

It is generally believed that leakage of the blood spinal cord barrier plays a crucial role in I-R injury [4]. Damage to the blood-spinal cord barrier may induce spinal cord edema, which can damage the spinal cord or even cause death [5]. Therefore, procedures that decrease blood spinal cord barrier permeability after I-R injury would improve the prognosis for patients with spinal cord I-R injury.

In animal models, ischemic preconditioning (IPC) has been reported to minimize I-R injury in many organs, including the spinal cord. The mechanisms that have been alleged to be responsible for IPC include: (1) improving local spinal cord blood flow and tissue oxygenation [6]; (2) enhancing the binding profile of heat shock proteins [7]; and (3) suppression of apoptosis [8]. Although IPC has been shown to attenuate ischemic brain edema and cerebrovascular injury in rats [9], there are limited studies regarding the effects of IPC on blood spinal cord barrier permeability after I-R injury.

Tight junctions are the structural and functional basis of blood brain barrier integrity, and the tight junction associated protein zonula occluden-1 (ZO-1) has been used as a tight junction marker protein [10]. After I-R injury, dissociation of ZO-1 from the cytoskeletal complex is frequently associated with increased blood brain barrier permeability [11]. Matrix metalloproteinases (MMPs) and tumor necrosis factor-α (TNF-α) also play a critical role in blood brain barrier and blood spinal cord barrier disruption in pathological conditions. For example, MMP-9 plays a key role in abnormal vascular permeability and inflammation immediately after a spinal cord injury. Blocking MMP-9 activity decreases vascular permeability and improves functional recovery [12]. TNF-α contributes to the opening of the blood brain barrier by a mechanism involving soluble guanylyl cyclase and protein tyrosine kinase [13].

The purpose of this study was to examine the protective effects and underlying mechanisms of IPC on blood spinal cord barrier permeability.

## 2. Results and Discussion

### 2.1. IPC Improves Neurologic Scores after Spinal Cord I-R Injury

Spinal cord I-R injury is a potentially devastating and unpredictable complication of thoracic aorta aneurysm surgery. Occlusion of the descending thoracic aorta reduces spinal blood flow in many animal models and in humans [3]. Several protective strategies have been developed that attempt to preserve blood supply or increase spinal cord ischemic tolerance and thereby prevent paraplegia [14–17]. Unfortunately, no single clinical intervention has been shown to be completely effective [15].

**Figure 1:**
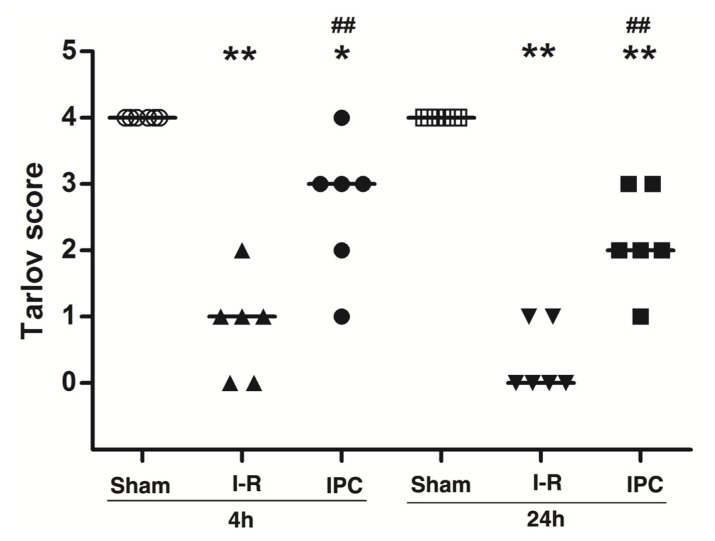
IPC improved neurologic function after spinal cord ischemia-reperfusion (I-R) injury. Neurologic function was assessed using Tarlov scores at 4 and 24 h after spinal cord ischemia. Data are presented as individual values for each animal, as well as median values for each group (*n* = 6/group). **p < 0.01 *versus* Sham group. ^##^*p* < 0.01 *versus* I-R group.

IPC involves brief ischemic episodes followed by periods of reperfusion and has been shown to protect against further ischemic damage [6]. The current study demonstrated that 30-min aortic occlusion leads to a high incidence of complete paraplegia in this animal model. However, IPC significantly improved the animal’s neurologic status after an I-R injury. Tarlov neurologic scores at 4 and 24 h after reperfusion are shown in Figure 1. Animals in the Sham group maintained normal motor function throughout the study. A 30 min aortic occlusion caused severe lower-extremity neurologic deficits in the I-R group at 4 and 24 h after reperfusion (*p* < 0.01). IPC protected against I-R, as the IPC group had better motor function than the I-R group at the 4 h (*p* < 0.01) and 24 h follow-up evaluations (*p* < 0.01).

### 2.2. IPC Inhibits Blood Spinal Cord Barrier Breakdown after Spinal Cord I-R Injury

Under physiological conditions, the blood brain barrier and blood spinal cord barrier represent a tight barrier between the circulating blood and central nervous system, formed by dense tight junction proteins, which seal the space between adjacent brain endothelial cells. Disruption of the blood brain barrier occurs under various pathological conditions, such as stroke and spinal cord injury, leading to an increased cerebrovascular permeability with subsequent development of tissue edema [5]. In the present study, IPC significantly attenuated the effects on I-R injury on blood spinal cord barrier permeability and spinal cord edema at both 4 and 24 h after injury.

**Figure 2:**
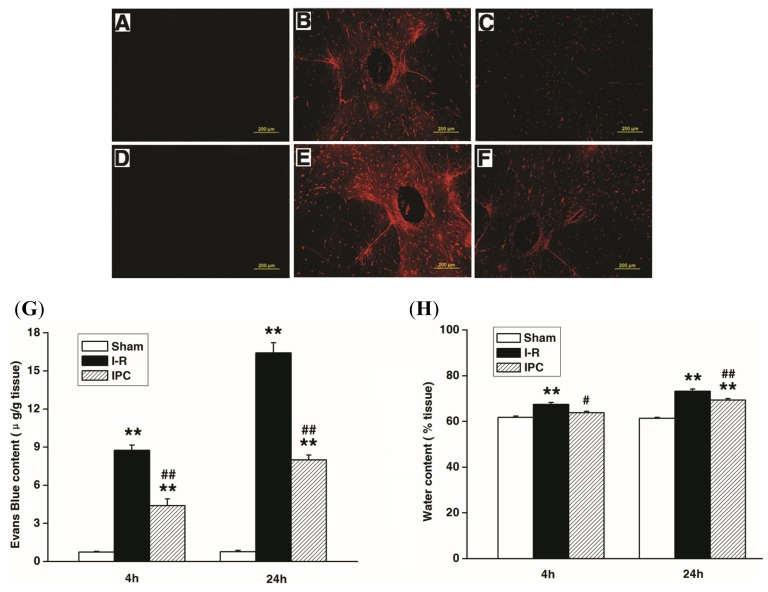
IPC inhibited the blood spinal cord barrier breakdown after spinal cord ischemia-reperfusion (I-R) injury. Blood spinal cord barrier permeability was measured at 4 and 24 h after injury using Evan’s Blue dye, and spinal cord edema was measured at 4 and 24 h by tissue water content. (**A**–**F**) Representative fluorescence images of Evan’s Blue extravasation in spinal cord parenchyma across groups: (**A**) Sham 4 h; (**B**) I-R 4 h; (**C**) IPC 4 h; (**D**) Sham 24 h; (**E**) I-R 24 h; (**F**) IPC 24 h; (**G**) Quantification of the content of Evan’s Blue in the injured spinal cord; (**H**) Quantification of the water content of the spinal cord. All data represent mean ± SEM (*n* = 6/group). **p < 0.01 *versus* Sham group. ^#^*p* < 0.05, ^##^*p* < 0.01 *versus* I-R group.

Evan’s Blue tracer is commonly used to evaluate the vascualr permeability. As shown in Figure 2, spinal cord I-R injury caused a marked increase in the amount of Evan’s Blue dye extravasation compared with sham controls, implying blood spinal cord barrier leakage. Furthermore, IPC treatment significantly reduced the amount of extravasation at 4 and 24 h after injury compared with the I-R group (Figure 1A–F). Quantitative analysis confirmed that I-R increased extravasation, while IPC attenuated this effect (*p* < 0.01) (Figure 2G). Assessment of water content showed similar results, as I-R increased water content due to spinal cord edema (*p* < 0.01), while IPC attenuated this effect at 4 h (*p* < 0.05) and 24 h (*p* < 0.01), as shown in Figure 2H.

### 2.3. IPC Preserves Tight Junction Protein ZO-1 after Spinal Cord I-R Injury

Tight junctions are the major structure responsible for restricting paracellular escape of compounds across the cerebral endothelium [18]. When tight junction integrity is disrupted, such as after cerebral I-R injury, transport of compounds across the blood brain barrier increases [10]. ZO-1, the main tight junction associated protein, plays an important role in connecting transmembrane and cytoskeleton proteins [19].

**Figure 3:**
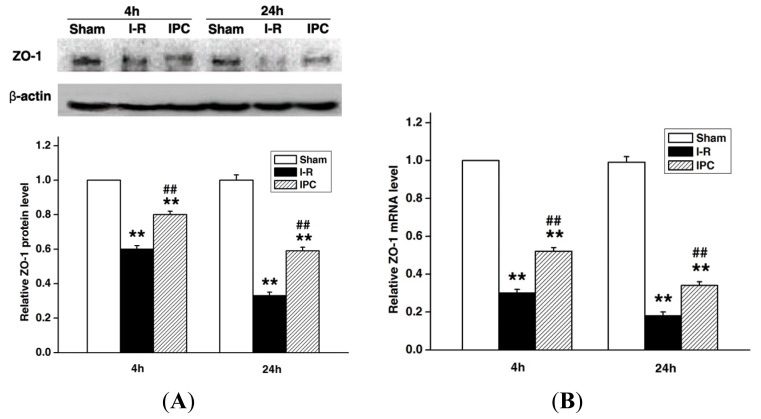

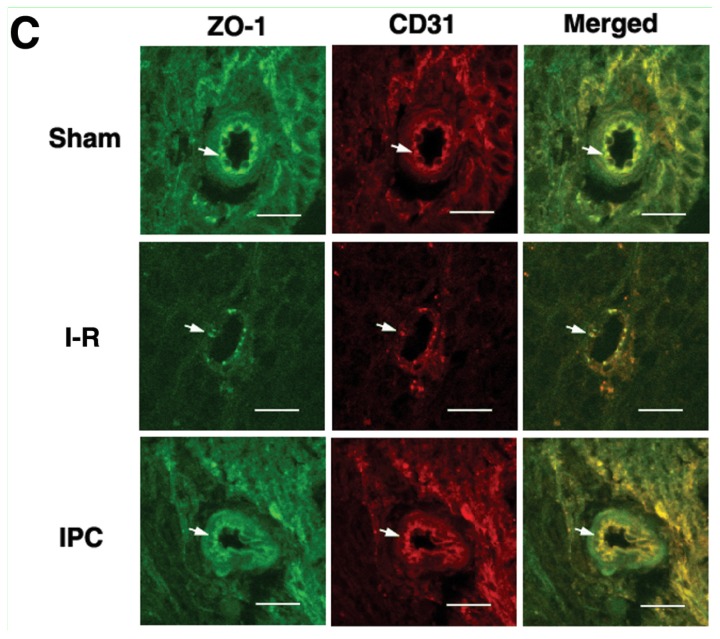
IPC increases expression of tight junction protein ZO-1 after spinal cord ischemia-reperfusion (I-R) injury. (**A**) Representative Western blot and quantitative protein analysis of ZO-1 expression in spinal cord at 4 h and 24 h after injury; (**B**) Real-time PCR analysis of ZO-1 mRNA expression in spinal cord at 4 and 24 h after injury. Levels are expressed as ratios to sham. Data are presented as mean ± SEM (*n* = 6/group). **p < 0.01 *versus* Sham group. ^##^*p* < 0.01 *versus* I-R group; (**C**) Representative double-labeling immunofluorescence microscopic photographs show that CD31-positive endothelial cells (red) express ZO-1 (green) at 24 h after injury. Bar scale = 20 μm.

To investigate the role of ZO-1 in IPC-induced protection of blood spinal cord barrier integrity, expression levels were examined by Western blotting and real-time PCR. As shown in Figure 3, ZO-1 levels were decreased at 4 and 24 h after spinal cord I-R injury, and IPC treatment significantly attenuated this effect at both time points (*p* < 0.01). Double-labeling immunofluorescence for ZO-1 with CD31 (vascular endothelial cell marker) revealed that I-R injury led to a decrease and discontinuous arrangement of ZO-1-positive protein along the microvasculatures compared to sham controls, and IPC once again attenuated this effect. These data indicate that IPC preserves blood spinal cord barrier integrity after spinal cord injury by inhibiting the degradation of the tight junction molecule ZO-1.

### 2.4. IPC Inhibits MMP-9 and TNF-α Expression after Spinal Cord I-R Injury

MMP-9 plays a critical role in blood brain/spianl cord barrier disruption in pathological conditions by degrading crucial components of cerebrovascular matrix, including collagen, laminin, and tight junction proteins, such as ZO-1 [20]. Upregulation of MMP-9 after spinal cord injury degrades components of the blood spinal cord barrier and facilitates immune cell infiltration, processes that have been implicated in secondary damage to the spinal cord [12].

In a previous study, cerebral I-R injury induced a significant increase in TNF-α levels at 3 and 72 h after reperfusion, accompanied by reduced ZO-1 expression [21]. In addition, TNF-α stimulated pericytes to release MMP-9, which lead to disruption of the blood brain barrier [22].

**Figure 4:**
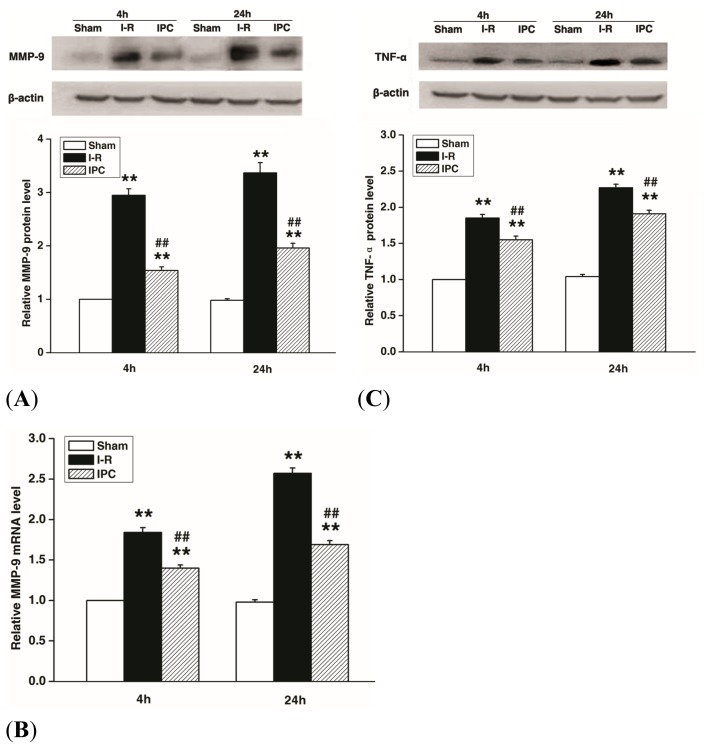
IPC inhibits MMP-9 and TNF-α expression after spinal cord ischemia-reperfusion (I-R) injury. (**A**) Representative Western blot and quantitative protein analysis of MMP-9 in the spinal cord at 4 and 24 h after injury; (**B**) Real-time PCR analysis of MMP-9 mRNA expression in the spinal cord at 4 and 24 h after injury; (**C**) Representative Western blot and quantitative protein analysis of TNF-α in spinal cord at 4 and 24 h after injury. Levels are expressed as ratios to Sham group. Data are presented as mean ± SEM (*n* = 6/group). **p < 0.01 *versus* Sham group. ^##^*p* < 0.01 *versus* I-R group.

In the current study, IPC decressed MMP-9 and TNF-α expression induced by spinal I-R injury. As shown by Western blots and real-time PCR, MMP-9 expression was markedly increased at 4 and 24 h after I-R injury (*p* < 0.01). IPC significantly decreased MMP-9 expression compared to I-R controls at both time points (*p* < 0.01) (Figure 4A,B). TNF-α expression showed a similar increase after I-R, which was attenuated by IPC (*p* < 0.01) (Figure 4C).

There are some acknowledged limitations to the present study. Only one IPC model and one ischemic time period was studied. The 30-min period of ischemia was chosen based on findings in earlier studies [8,23,24]. Since longer periods of ischemia may lead to different outcomes with respect to the protective effects of IPC against I-R injury, the selection of a 30 min aortic cross clamp time can be considered a limitation of the present study. Another limitation in this study arises from the time window to observe blood spinal cord barrier changes. It was reported that ischemic preconditioning protected the spinal cord against neuronal damage 24 h but not at 7 days after an I-R injury [25]. Meanwhile, breakdown of blood spinal cord barrier peaked within 24 h after spinal cord I-R injury [26]. Thus, we focused on blood spinal cord barrier integrity at 4 and 24 h after I-R injury. A longer observation time would be important in future studies assessing the protective effects of IPC on blood spinal cord barrier after an I-R injury. Though spinal cord ischemia preconditioning has not been used in clinical at present, the protective effects of IPC against spinal cord ischemia injury had been clarified in various animal models [6,7,27] and its operation process is completely in line with thoracoabdominal aortic aneurysm operation. It is thus clear that IPC will be a promising strategy to protect spinal cord in the near future.

## 3. Experimental Section

### 3.1. Animal Care and Experimental Protocol

Japanese white rabbits, weighing 2.0–2.3 kg, were obtained from the animal center of the China Medical University. Animals were housed with free access to food and water. All experimental procedures were approved by the Ethics Committee of China Medical University and were in accordance with the NIH Guide for the Care and Use of Laboratory Animals. Animals were randomly divided into three groups. The I-R group underwent a 30 min aortic occlusion followed by reperfusion. The IPC group underwent three cycles of 5 min aortic occlusion followed by 5 min reperfusion for ischemic preconditioning, and then I-R procedures. The Sham group underwent anesthesia and surgical preparation without ischemic preconditioning or I-R.

### 3.2. Surgical Procedure

Animals were anesthetized with 20% urethane (i.v.) at an initial dose of 1 g/kg. Animals were allowed to breathe room air spontaneously. Body temperature was continuously monitored with a rectal probe and was maintained at 38.5 ± 0.5 °C with the aid of a heated operating table. Two catheters were inserted into the ear and femoral artery to measure proximal and distal blood pressure (Spacelabs Medical, Inc., Redmond, WA, USA). Following a midline laparotomy, the abdominal aorta was carefully exposed. Heparin 200 U/kg, i.v. was administered 5 min before occlusion. A bulldog clamp was then placed across the aorta, just 1 cm below the left renal artery, to produce abdominal aortic occlusion according to the method described previously [8,24].

### 3.3. Neurologic Assessment

At 4 and 24 h after reperfusion, an observer blind to the treatment group assessed motor function using the modified Tarlov criteria as follows: 0 = paraplegic with no lower extremity function; 1 = poor lower extremity function, weak antigravity movement only; 2 = some lower extremity function with good antigravity strength, but inability to draw legs under body; 3 = ability to draw legs under body and hop, but not normally; and 4 = normal motor function [28].

### 3.4. Measurement of Blood Spinal Cord Barrier Permeability

Blood spinal cord barrier disruption was measured by extravasation of Evan’s blue as previously described [29]. Briefly, 2% Evan’s Blue (10 mL/kg, i.v.) was injected and the animals were kept anesthetized for 30 min. The animals were then perfused with 0.9% saline containing 10 U/mL heparin. The L4-L6 spinal cord segment was removed and weighed, then homogenized in methanamide (1 mL/100 mg). Homogenized samples were stored at 60 °C for 24 h before centrifugation at 20,000× *g* for 20 min. Absorbance was measured at 632 nm using a spectrophotometer (BioTek, Winooski, VT, USA). Data are reported as the amount of Evan’s Blue per wet tissue weight (μg/g). For qualitative examination of extravasations, animals were perfused with 0.9% saline containing 10 U/mL heparin, and then the spinal cord was fixed by 4% paraformaldehyde. Spinal cords were cut in 10 μm sections using a cryostat, and Evan’s Blue fluorescence was observed under a fluorescence microscope (Olympus BX-60, Tokyo, Japan).

### 3.5. Measurement of Spinal Cord Edema

The water content of the spinal cord was measured by a wet-dry method to provide a quantitative measure of edema. Animals were anesthetized and the L4-L6 spinal cord segment was immediately removed and weighed, then dried at 110 °C for 24 h and reweighed. Percent water content was calculated as: (wet weight − dry weight)/wet weight × 100.

### 3.6. Immunofluorescence of Tight Junction Protein ZO-1

To measure the expression of the tight junction protein ZO-1 in vascular endothelial cells, double immunofluorescence staining of ZO-1 and the endothelial cell marker CD31 was used. Rabbits were decapitated after perfusion with 0.9% saline and 4% paraformaldehyde at 4 and 24 h after reperfusion. After a 24 h fixation, the L4-L6 spinal cords were frozen and 10-μm-thick sections were processed for immunofluorescence staining. Sections were placed on slides, fixed for 5 min in ice-cold acetone, and blocked with 10% bovine serum albumin for 1 h at room temperature. Subsequently, sections were incubated with primary mouse anti-CD31 antibody (1:20, Abcam, Cambridge, MA, USA) overnight at 4 °C. Sections were then washed three times for 5 min in PBS and incubated with donkey anti-mouse Alexa fluor 594 (1:500, Invitrogen, Camarillo, CA, USA) for 1 h at room temperature. Sections were incubated by mouse anti-ZO-1-FITC conjugate antibody (1:50, Invitrogen, Camarillo, CA, USA) for 1 h at room temperature. Images were captured using a Leica TCS SP2 (Leica Microsystems, Buffalo Grove, IL, USA) laser scanning spectral confocal microscope.

### 3.7. Western Blots

Western blots were performed as previously described [30]. The rabbits’ spinal cords were homogenized, and total proteins were purified using cell and tissue protein extraction reagents according to manufacturer’s instructions (KC-415; KangChen, Shanghai, China). In all, 30 μg protein equivalent of each sample was electrophoresed on a polyacrylamide gel, transferred onto nitrocellulose, and probed with monoclonal anti-ZO-1 antibody (Invitrogen, Camarillo, CA, USA), anti-MMP-9 antibody (Abcam, Cambridge, MA, USA), or anti-TNF-α antibody (Bioss, Beijing, China), followed by horseradish peroxidase (HRP)-conjugated secondary antibodies (Bioss, Beijing, China). Semi-quantization of scanned films was performed using Quantity One software (Bio-Rad, Segrate, Italy).

### 3.8. Quantitative Real-Time Polymerase Chain Reaction Analysis

The mRNA levels of ZO-1 and MMP-9 were measured by real-time polymerase chain reaction (PCR) analysis following a standard method with minor modifications [31]. Briefly, total RNA was extracted using a TRIzol Kit according to manufacturer’s instructions. cDNA was prepared using a Prime Script RT Reagent kit (Takara, Dalian, China) and quantitative real-time PCR was performed using an ABI 7000 instrument (Applied Biosystems, Foster City, CA, USA) and a SYBR Green Real-time PCR Master Mix (Toyobo, Osaka, Japan). Amplification was performed as following: 50 °C for 2 min (UDG incubation), 95 °C for 10 min, followed by 40 cycles of denaturing at 95 °C for 15 s and annealing at 60 °C for 30 s. All reactions were performed in triplicate. Melting curve analysis was performed to ensure the specificity of quantitative PCR. Data analysis was performed using the 2(-Delta Delta C(T)) as previous described [32], where GAPDH was used as reference gene. Specific primers used for this quantification were as follows: ZO-1 (259 bp): forward 5′-CGT AAC ACC AAA TGC AGT AGA TCG TC-3′; reverse 5′-CTG TTG CTG GAT TGC TTC CTT CAA C -3′. MMP-9 (119 bp): forward 5′-TGT GTC TTC CCC TTC GTC TT-3′; reverse 5′-CCC CAC TTC TTG TCG CTG T-3′. GAPDH (177 bp): forward 5′-TCG GCA TTG TGG AGG GGC TC-3′; reverse 5′-TCC CGT TCA GCT CGG GGA G-3′.

### 3.9. Statistical Analysis

Data were presented as mean values ± SEM or median values (for neurologic assessments) and analyzed with Kruskal-Wallis test followed by the Mann-Whitney U test with Bonferroni correction. A *p* value of <0.05 was considered statistically significant.

## 4. Conclusions

In conclusion, the current study demonstrates that IPC can reduce blood spinal cord barrier permeability induced by spinal cord I-R injury in an animal model. Mechanisms underlying IPC-induced neuroprotection of the blood spinal cord barrier integrity include preservation of tight junction protein ZO-1 and downregulation of MMP-9 and TNF-α.
